# Minor Aids to Medical Study

**Published:** 1888-06-23

**Authors:** 


### Minor Aids to Medical Study.

Among the greater helps to the acquirement of medical knowledge and skill accomplished teachers and well-written books occupy a conspicuous place; among the smaller aids good reading-rooms and comfortable chairs are not to be despised. No doubt a thoroughly determined student will acquire knowledge and fight his way to skill whatever difficulties may stand in his way. To him obstructions are made for no other purpose but to be removed or beaten down. But even in the case of the ablest, diminished facility means less perfect efficiency, whilst helps of all kinds lead to higher standards of perfection. How few, however, belong to the ranks of the ablest! The world consists of a large proportion who are moderately capable; of a larger proportion who can only be made capable by good training; and of a small sprinkling of those who will make themselves capable. He is a poor philosopher who quarrels with the world because it does not consist of able men, an unpractical statesman who refuses to reckon with and provide for the innumerable ranks of the commonplace.

All this is *apropos* of chairs, tables, and a large sitting-room which have been provided for medical students at the Westminster Hospital by the good sense of the Managing Committee. The students' hall, or common room, and their cloak-room have been newly furnished and made comfortable throughout, greatly to the satisfaction of the students, and in furtherance of their well-being. No doubt people who hail from the country and who imagine that in these luxurious days everything of the pleasantest and the most costly is at the disposal of the young gentlemen who condescend to study physic, will be a little surprised to be told that a common hall is but now made thoroughly comfortable at a large metropolitan hospital, and that students are seriously congratulating themselves thereupon. Nevertheless, such is the fact. At many a metropolitan hospital a very short time ago comfortable reading-rooms and modest facilities for study of that elementary kind were comparatively unknown. How could it be otherwise? Hospitals are supported by the money of the charitable. The charitable, it was thought, did not give their money to be spent on chairs, tables, and softly-carpeted floors for budding doctors. No, indeed! But we have come to take rather a different view of hospitals from what was formerly taken. They used to be places where the sick were tended and cured. That was their chief and almost their only function. They are still places where the sick are tended and cured, but they are also places where the science and art of medicine are thoroughly taught; they are indeed the only places where it can be thoroughly taught; and the only places therefore from whence the whole country can be supplied with fully-equipped and competent medical men.

The public are much more interested in making medical students comfortable and encouraging them by all possible means to study diligently than hospital patients are. Hospitals and their patients can always command the best medical and surgical talent; the public must take the average. Whatever depresses this average lowers the status and skill of the ordinary practitioner who serves the public; whatever raises, increases his learning and efficiency. The The public is therefore immediately and supremely interested in placing in the way of hospitals, their teachers and students, every facility both great and small, both costly and inexpensive, which makes the hospital and its work more attractive to the student. All people of enlightened self-interest as well as those who have the virtue to rise above the question of self-interest, will applaud the action of the Westminster Committee. Any who take a special interest in this hospital, or in medical students will do well to visit and inspect the improvements for themselves. For this purpose Tuesday is the best day. It is only necessary to go on any Tuesday and send a card to the secretary to ensure all possible civility and attention.

